# Mapping Evidence on Community-Based Clinical Education Models for Undergraduate Physiotherapy Students: Protocol for a Scoping Review

**DOI:** 10.2196/19039

**Published:** 2020-10-20

**Authors:** Nomzamo Charity Thobekile Chemane, Verusia Chetty, Saul Cobbing

**Affiliations:** 1 Department of Physiotherapy University of Kwa Zulu Natal Durban South Africa

**Keywords:** physiotherapy, clinical education, community-based clinical training, decentralized clinical training, primary health care

## Abstract

**Background:**

Community-based clinical training has been advocated as an excellent approach to transformation in clinical education. Clinical education for undergraduate physiotherapy students is a hands-on practical experience that aims to provide a student with the skills necessary to enable them to be fit to practice independently. However, in many countries, including South Africa, this training has been conducted only in large urban academic hospitals. Such hospitals are not a true reflection of the environment that these students will most likely be facing as practicing health care professionals.

**Objective:**

The objective of this scoping review is to map out existing evidence on community-based clinical education models for undergraduate physiotherapy students globally.

**Methods:**

A systematic scoping review will be based on the 2005 Arksey and O’Malley framework. Studies involving students and stakeholders in clinical education will be included. This review will not be limited by time of publication. An electronic search of relevant literature, including peer-reviewed primary studies and grey literature, will be conducted from the PubMed, Google Scholar, Medline, CINAHL, and Cochrane Library databases. The search strategy will include keywords such as “education,” “physiotherapy,” “undergraduate,” “community-based,” “training,” “decentralized,” and “distributed.” Boolean logic will be used for each search string. Two independent reviewers will conduct screening of titles, abstracts, and full text before extracting articles. A predesigned data-charting table will supplement the extraction of data. Version 12 NVIVO software will aide in the thematic analysis of data.

**Results:**

Data collection will commence after publication of this protocol, and the results are expected to be obtained in the following 5 months.

**Conclusions:**

The evidence obtained from the extracted data is expected to assist in the development of a model of community-based clinical education for undergraduate physiotherapy students in South Africa, and serve as a basis for future research. The discussion of this evidence will be guided by the research question utilizing a critical narrative approach to explore emerging themes. The enablers and barriers identified from the reviewed studies can guide the development of a community-based clinical education model.

**International Registered Report Identifier (IRRID):**

PRR1-10.2196/19039

## Introduction

Clinical education is the integration of theory into practice in the health care environment, with the aim of developing clinically competent health science practitioners. This type of training is imperative in health science professions as it provides hands-on practical experience with real patients in a real clinical environment [1-3]. Traditionally, this training has been centered around well-resourced academic hospitals, mainly in cities close to universities [4]. However, a global shift toward community-based clinical practice conducted away from a university setting and large academic hospitals necessitates a change toward rural and periurban clinical placements [5]. This type of training is also known as decentralized clinical training [6,7].

The primary health care approach has been identified as a “first level of contact in a health system” [8]. Therefore, it is essential for a curriculum to address the primary health care needs of the population, which are social responsiveness, inclusiveness, and participation [9]. This transformation prepares undergraduate students to be socially responsive to the needs of the communities they serve, giving them the confidence to become health advocates for their patients [4,10,11].

Physiotherapy is one of the few health science professions that manages patients from the acute hospital phase in the intensive care unit to a chronic rehabilitation phase in the primary health care setting in the communities in which patients live. Therefore, diversified clinical training of physiotherapy students is essential for a curriculum that aims to provide clinical competence and social accountability [3,12-15]. Although this is also the case for medical students [6,16], there have been extensive global debates regarding practical placements and their effectiveness in producing graduates who are prepared for the changing health needs in the developing world [5,13,17-19]. The primary purpose of a school of physiotherapy is to develop graduates that have both the clinical reasoning and practical skills required to function as competent practitioners in all levels of care [20].

Global research on health education programs [2,3,18,19,21,22] concurs that a community-based clinical training program that uses decentralized clinical training platforms is an excellent approach. This approach aims to achieve transformative learning, which enhances ethical and social accountability [17]. Decentralized clinical training in this context is defined as training closer to the community, away from universities and large academic hospitals. The changes in health education, including an increase in student intake and health systems requirements, are a driving force to ensure that students are well prepared to meet the demands of their communities. This will require an improvement, review, or change in the curriculum to ensure the preparedness of graduates to be competent professionals who can implement knowledge, skills, and values practically [3,7].

A scoping literature review conducted by De Villers et al [6] confirmed that medical training in sub-Saharan Africa conducted at different clinical settings distant from large academic hospitals is beneficial in improving core competencies for students and in retaining these graduates in rural settings. However, less is known about other health science undergraduate programs in this regard, specifically for the discipline of physiotherapy. Therefore, there is a need for evaluation of existing global community-based clinical education models to contribute toward the development of a community-based primary health care training model in the South African context. This scoping review aims to examine and map evidence related to community-based clinical education models for undergraduate physiotherapy students and highlight their ability to produce socially responsive graduates. The results of this review will contribute toward the development of a community-based primary health care training model for undergraduate physiotherapy students in the South African context.

## Methods

### Study Design

The methodology for this scoping review will adopt the five-stage framework developed by Arksey and O’Malley [23], which Levac and colleagues [24] further elaborated by including aspects of quality appraisal. These stages are described in more detail below, in specific relation to the primary aim of this study. A Preferred Reporting Items for Systematic reviews and Meta-Analyses (PRISMA) extension for a scoping review checklist (Multimedia Appendix 1) will be used to ensure the inclusion of all relevant sections.

### Stage 1: Identifying the Research Question

The main question that will guide this review is, “What are existing models of community- based clinical education for undergraduate physiotherapy students?”

The subsequent subquestions that will pave the way for the review are as follows: (1) What are the clinical education models that exist for the physiotherapy discipline? What is/has been the practice? (2) How have community-based clinical education models been put to practice? (3) What are the enablers and barriers of the identified clinical education models? (4) Does any clinical education model utilize decentralized training platforms to ensure clinical competence through community engagement and social learning?

The Participants-Concept-Context model will be adopted to determine the eligibility of the research question (Table 1) [25].

**Table 1:** Participants-Concept-Context framework for eligibility of the research question.

Components	Determinants
Participants	Physiotherapy students at the undergraduate level of study, academics, clinical supervisors
Concept	Models of community-based clinical education
Context	Global

### Stage 2: Identifying Relevant Literature

A comprehensive search strategy will be developed for this review to harness related studies. The electronic databases searched will include PubMed, Pedro, MEDLINE and CINAHL, Google Scholar, an academic search using EBSCOhost via the University of Kwa-Zulu Natal (UKZN), and Cochrane Library. Keywords will be separated by Boolean terms “AND,” “OR,” “NOT.” The final step will be the search of the reference lists. The initial list of keywords will include, but are not limited to: “clinical education,” “training,” “teaching and learning,” “undergraduate physiotherapy education,” “decentralized” OR “distributed,” “community-based,” “community-engaged,” “primary health care,” “physiotherapy”/“physical therapy,” and “curriculum.” A pilot study was conducted to determine the feasibility of the study. The pilot findings showed good feasibility of the study with 118 articles retrieved from PubMed and 16,616 articles obtained from EBSCOhost (Table 2).

**Table 2:** Results of a pilot search.

Keywords searched	Database	Date of search	Number of publications retrieved
((((((“Physiotherapy” OR “Physical Therapy”)) AND (“Training” OR “Education” OR “Teaching” OR “Teaching and Learning” OR “Curriculum”))) OR (((“education”) AND “physiotherapy”) AND “undergraduate” AND (Humans[Mesh])) AND (Humans[Mesh]))) OR (((“Decentralized” OR “Distributed” OR “Community- based” OR “Community engaged”)) OR “on the job” AND rural AND”) OR “ AND primary health care AND (Humans[Mesh])) AND (Humans[Mesh])	PubMed	September 27, 2019	118
((((((“Physiotherapy” OR “Physical Therapy”)) AND (“Training” OR “Education” OR “Teaching” OR “Teaching and Learning” OR “Curriculum”))) OR (((“education”) AND “physiotherapy”) AND “undergraduate” AND (Humans[Mesh])) AND (Humans[Mesh]))) OR (((“Decentralized” OR “Distributed” OR “Community- based” OR “Community engaged”)) OR “on the job” AND rural AND) OR AND “primary health care” AND (Humans[Mesh])) AND (Humans[Mesh])	EBSCOhost	September 27, 2019	16,616, including the following filters: human, (full text, scholarly (peer-reviewed) journal

### Stage 3: Study Selection

The study research question will be utilized to guide the development of the inclusion and exclusion criteria for the proper selection of relevant studies.

Peer-reviewed articles published in English that focus on the following theory will be included: (1) models of undergraduate physiotherapy community-based clinical education, (2) undergraduate physiotherapy curricula on clinical education, and (3) decentralized clinical training (ie, training conducted away from the university and central training academic hospitals, including rural sites, primary health clinics, community health centers, district hospitals, and regional hospitals).

Opinion papers on community-based clinical education for undergraduate physiotherapy students will be excluded, such as commentaries on community-based clinical training for undergraduate physiotherapy students.

### Charting of Data

A data-extracting tool will be created to organize and store all data retrieved from the articles during the scoping review. Two independent reviewers utilizing the sample of the included studies will evaluate this tool. The information from studies will consist of: author, year of publication, site location, study population, institution description (community health center, primary health clinic, hospital, community, home), site description (rural, periurban, or urban), duration of the training at the site, aim or purpose of the study, methodology, essential results, model aspects, and recommendations. This information will be continuously updated throughout the scoping review process. All eligible studies will be uploaded to Mendeley referencing software and replicate studies will be removed. PRISMA guidelines will be used to report the screening results [26].

### Collating, Summarizing, and Reporting Results

This review will adopt a mixed-method analysis of the results of the selected studies, including both qualitative and quantitative analyses. Extracted data that will be analyzed quantitatively will include numerical summaries of article type, duration of rotation, site description, location (rural, urban, periurban), and the aspects of the model. A descriptive analytical method will be conducted using the Statistical Package for Social Sciences Version 23. Thematic analysis will be used for analyzing the qualitative data from the reviewed studies to synthesize and interpret critical issues and themes arising from the included studies.

### Quality Appraisal

The Mixed Method Appraisal Tool (MMAT) version 2018 [25] will be used to appraise the quality of the selected studies, as recommended by Levac et al [24]. Three reviewers (NT, VC, and SC) will be involved in the critical appraisal process. Two reviewers will capture methodological quality criteria, according to MMAT [27]. A third reviewer who is an expert in MMAT application will oversee the complete process, adding rigor to the process. The MMAT allows for a concomitant appraisal of methodological quality of five study categories: qualitative research, randomized controlled trials, nonrandomized studies, quantitative descriptive studies, and mixed methods studies [25].

### Ethics Approval

The study is part of doctoral work in the Department of Health Sciences at UKZN. Ethical approval was obtained from the Humanities and Social Sciences Research Ethics Committee of UKZN (ethical clearance no. HSS/0575/018D).

## Results

Data collection will commence upon protocol publication, and the results can be expected in the following 5 months.

## Discussion

This scoping review aims to map out existing models of community-based clinical education and highlight their ability to produce socially responsive graduates. There is a global shift toward community-based clinical training of health care professionals with evidence supporting this approach in undergraduate medical education [19,22].

The undergraduate physiotherapy curriculum needs to produce graduates who possess the competencies of a health practitioner, professional, scholar, health advocate, collaborator, communicator, and leader. Decentralized clinical training has been reported as the best method of developing competent undergraduate students who will be socially accountable and able to advocate for their patients [6,28].

This scoping review will synthesize the evidence and reveal knowledge gaps to contribute toward the development of a community-based clinical education model for undergraduate physiotherapy students in a South African context.

Clinical education stakeholders, physiotherapist clinical supervisors in different hospital settings, and academics involved in the training of undergraduates stand to benefit from this scoping review. The review will produce consolidated evidence of various models of community-based clinical education for undergraduate students. This evidence can be employed by stakeholders to design future programs and also form a basis for future research.

This scoping review will clearly describe the global community-based clinical education models used for the training of undergraduate physiotherapy students. The empirical evidence obtained from this review will be beneficial to stakeholders in health science education, including academics, clinicians, and policymakers, contributing to the ongoing transformation of clinical training.

## Appendix

Multimedia Appendix 1 Preferred Reporting Items for Systematic reviews and Meta-Analyses extension for Scoping Reviews (PRISMA-ScR) checklist.

## Abbreviations

MMAT: Mixed Methods Appraisal Tool
PRISMA: Preferred Reporting Items for Systematic reviews and Meta-Analyses
UKZN: University of Kwa-Zulu Natal
